# Two Cases of Abdominal Section

**Published:** 1897-06

**Authors:** J. Raglan Thomas


### TWO CASES OF ABDOMINAL SECTION.

BY

J. Raglan Thomas, M.D. Lond., M.R.C.S. Eng.

The following cases seem to possess special features of sufficient interest to justify one in placing them on record.

### Large dermoid ovarian cyst: base surrounding uterus.

A married lady, aged 47, who had never been pregnant, had, two years ago, menorrhagia suddenly at one period only. A firm rounded tumour, feeling like a fibroid, could be felt reaching to about an inch above the pubes, slightly towards the right side. This had never been observed previously by the patient. Six months later catamenia ceased. No vaginal examination was allowed, and the case was lost sight of until lately. When seen again, towards end of 1896, the tumour had increased enormously. There had been no pain; but the growth was causing inconvenience and discomfort from its size. In February it reached nearly to the costal arch, was extremely tense, and fluctuation could be felt in front, and from the front to finger within vagina. The flanks outside the tumour were quite resonant. The skin moved over it slightly, and the mass could be pushed a little from side to side, but appeared to have a wide firm attachment within the pelvis. The uterus was normal in size and position, and fairly movable. To the left of this organ, in the ovarian region, could be felt a firm rounded mass, apparently attached to the tumour, with which it moved, whereas the uterus moved independently of it. This was taken to be the pedicle of the growth, which, it was thought, might probably be held down in the pelvis by adhesions.

In consequence of rapid increase towards the left hypochondrium, and great tenseness, operation, previously advised, was urged as imperatively necessary. This was performed by me in the ordinary way on March 4th, 1897. The walls of the cyst were thick and fleshy, except in front, where they were much stretched and thinned. About twelve pints of dirty cream-coloured fluid were drawn off, and the cyst, which contained also large balls of hair, teeth, and other dermoid material, was delivered, having no adhesions. It was then found that the base completely surrounded the uterus, the outlines of which could not be defined from within the peritoneal cavity. The right ovary and tube could not be distinguished. Those organs on the left side, much enlarged, with masses of glands, formed a long sausage-shaped roll, about 1 in. in diameter, attached to the side of the tumour and to the pelvic wall. This had been mistaken for the pedicle, which now was obviously more connected with the opposite side and with the whole of the uterus. The greater part of the walls were cut away, and the remainder of the cyst shelled out. It had been apparently divided by septa which had broken down. A small abscess, to the left of the base, was cleared of its viscid contents, and, as far as possible, cut and scraped away. The enlarged left ovary, tube, and glands were ligatured and removed. The stump, which, by its folds, had still a large peritoneal surface, was carefully stitched into the lower angle of the wound, leaving the raw surfaces exposed. After irrigation, a glass drainage tube was inserted, and the rest of the abdominal wound closed.

The patient did well, the temperature ranging from 97.4° to 99.4° for the first fortnight. Then some slight trouble arose at the site of the old abscess-cavity referred to, which was drained and syringed daily. This closed within a week, and the patient is now well, and able to walk out of doors.

### Removal of ovaries and uterine appendages for early cystic disease, &c.

A married lady, 37, had one child, nine years ago, whom she nursed six weeks. Four months later she began to feel ill with pain in the back, and leucorrhœa. Two years afterwards curetting was done with temporary benefit. Three years ago she began to notice swelling and pain in left ovarian region after nearly every period, usually culminating about ten days later, when "something seemed to give way," with a discharge of fluid *per vaginam*, sometimes clear, at others yellowish. Menstruation about every three weeks. Consequently she had to lie on a sofa or in bed half her time, and from leading a very active useful life, was gradually becoming a chronic invalid. She described her life as miserable, and said she would rather die than live thus.

A few days after the period a fulness could be detected, tender and and painful, in the left ovarian region. *Per vaginam* a lump, very tender to pressure, could be felt, behind the normal position of the ovary. Through the rectum this felt like an enlarged ovary, displaced backwards and adherent. The swelling varied considerably in size at different visits. On one of these, about the tenth day, it was so considerable that it seemed to occupy the greater part of the space to the left of the uterus, which was now fixed and somewhat pushed over. Next day, a discharge having meanwhile occurred, I found the uterus quite movable, and in normal position; but, unfortunately, I was never allowed to see the discharge. There was slight fulness and tenderness in the normal position of the right ovary. The sound passed 2½ in.

The patient was most anxious to risk operation in the hope of being cured, notwithstanding that an eminent London specialist had declined to advise it. After careful observation, and not without a fear of finding extensive adhesions deep in the pelvis, I recommended the operation, which I did on February 10th, 1897. This proved easier than I had expected. The adhesions found were not extensive, and the left ovary was easily separated and removed. It was considerably enlarged, and contained several cysts, varying from ¾ in. diameter to ¼ in. The Fallopian tube was not dilated, as I had expected, but had several thin-walled cysts of various sizes attached to it by pedicles of various lengths. The right ovary, though in normal position, was almost as large and similarly diseased. With a view to secure the best prospect of permanent cure, the tubes on both sides were removed with the ovaries.

The patient made a rapid recovery, her temperature not exceeding 99°. She feels quite well, and has been free from pain, though four menstrual periods would have occurred meanwhile.

Dr. Kanthack, of St. Bartholomew's Hospital, kindly cut sections of the organs for me, and reported "numerous cysts, some large, some small, lined by epithelium varying from flattened to columnar. Ovary highly vascular. Nothing malignant. Probably early cystic disease of ovary."

Having had a large number of these most difficult cases under my care at different times, I have been much impressed with the necessity for the greatest care in regard to their diagnosis and treatment. I have seen on the one hand healthy ovaries removed, without relief of symptoms, and on the other useful lives become more and more hopelessly invalid and bed-ridden, in ever-recurring pain and sickness—a truly miserable existence—where operation in time might probably have effected a complete cure.

